# Therapeutic laquinimod treatment decreases inflammation, initiates axon remyelination, and improves motor deficit in a mouse model of multiple sclerosis

**DOI:** 10.1002/brb3.174

**Published:** 2013-09-23

**Authors:** Spencer Moore, Anna J Khalaj, JaeHee Yoon, Rhusheet Patel, Gemmy Hannsun, Timothy Yoo, Manda Sasidhar, Leonardo Martinez-Torres, Liat Hayardeny, Seema K Tiwari-Woodruff

**Affiliations:** 1 Multiple Sclerosis Program, Department of Neurology, David Geffen School of Medicine at UCLALos Angeles, California; 2 Brain Research Institute, UCLA School of MedicineLos Angeles, California; 3 Intellectual Development and Disabilities Research Center, UCLALos Angeles, California; 4 Pharmacology Unit, Global Innovative R&D, Teva Pharmaceutical IndustriesNetanya, Israel

**Keywords:** Axon conduction, demyelination, experimental autoimmune encephalomyelitis, laquinimod, multiple sclerosis, myelination, neuroprotective drug, oligodendrocytes, peripheral cytokines, rotorod motor performance, therapeutic treatment

## Abstract

**Background:**

Therapeutic strategies that induce effective neuroprotection and enhance intrinsic repair mechanisms are central goals for future treatment of multiple sclerosis (MS), as well as other diseases. Laquinimod (LQ) is an orally administered, central nervous system (CNS)-active immunomodulator with demonstrated efficacy in MS clinical trials and a favorable safety and tolerability profile.

**Aims:**

We aimed to explore the pathological, functional, and behavioral consequences of prophylactic and therapeutic (after presentation of peak clinical disease) LQ treatment in the chronic experimental autoimmune encephalomyelitis (EAE) mouse model of MS.

**Materials and methods:**

Active EAE-induced 8-week-old C57BL/6 mice were treated with 5 or 25 mg/kg/day LQ via oral gavage beginning on EAE post-immunization day 0, 8, or 21. Clinical scores and rotorod motor performance were assessed throughout the disease course. Immune analysis of autoantigen-stimulated splenocytes, electrophysiological conduction of callosal axons, and immunohistochemistry of white matter-rich corpus callosum and spinal cord were performed.

**Results:**

Prophylactic and therapeutic treatment with LQ significantly decreased mean clinical disease scores, inhibited Th1 cytokine production, and decreased the CNS inflammatory response. LQ-induced improvement in axon myelination and integrity during EAE was functional, as evidenced by significant recovery of callosal axon conduction and axon refractoriness and pronounced improvement in rotorod motor performance. These improvements correlate with LQ-induced attenuation of EAE-induced demyelination and axon damage, and improved myelinated axon numbers.

**Discussion:**

Even when initiated at peak disease, LQ treatment has beneficial effects within the chronic EAE mouse model. In addition to its immunomodulatory effects, the positive effects of LQ treatment on oligodendrocyte numbers and myelin density are indicative of significant, functional neuroprotective and neurorestorative effects.

**Conclusions:**

Our results support a potential neuroprotective, in addition to immunomodulatory, effect of LQ treatment in inhibiting ongoing MS/EAE disease progression.

## Introduction

Inflammation-triggered neurodegenerative damage is ultimately responsible for the primary and secondary progressive phases of multiple sclerosis (MS), and this damage is particularly refractory to currently approved immunomodulatory or immunosuppressive therapies (Bates 2011; Dutta and Trapp 2011). Laquinimod (LQ) is a once-daily oral immunomodulatory agent with potential neuroprotective properties. In Phase II clinical trials, LQ demonstrated a favorable safety profile and significantly reduced disease activity by decreasing the number of active lesions (Polman et al. 2005; Comi et al. 2008). In Phase III ALLEGRO and BRAVO clinical studies, LQ reduced annual relapse rate, significantly reduced disability progression and brain atrophy by 35%, and reduced the risk of sustained disability (Comi et al. 2012). More recently, LQ was found to increase levels of brain-derived neurotrophic factor (BDNF) in the serum of MS patients (Thone et al. 2012).

Experimental autoimmune encephalomyelitis (EAE) is one of the best mouse models of MS, and it has been used to understand neurodegenerative mechanisms in the setting of immune-mediated demyelination (Bannerman et al. 2005; Steinman and Zamvil 2006; Jones et al. 2008). The EAE model has been used extensively to elucidate immune mechanisms of currently approved MS drugs (Gasperini and Ruggieri 2009). Through its ability to reduce infiltrating cells in the central nervous system (CNS), LQ treatment within the EAE model has shown promising results by decreasing spinal cord demyelination and axonal loss (Brunmark et al. 2002; Yang et al. 2004; Gurevich et al. 2010; Wegner et al. 2010; Aharoni et al. 2012; Bruck et al. 2012). In addition, LQ treatment was shown to alter monocytes to a Type II phenotype, which direct T cells toward an anti-inflammatory response (Thone et al. 2012).

In this study, we aimed to further explore the pathological, functional, and behavioral consequences of prophylactic and therapeutic (after presentation of peak EAE clinical disease) LQ treatment in EAE mice. Detailed assessment of peripheral immune cell cytokine response was used to assess peripheral immunomodulation by LQ, while immunohistochemistry was used to assess immune cell infiltration into the CNS, axon health, and axon myelination. We and others have shown that CNS structures other than the spinal cord are negatively affected during EAE, leading to sensory, motor, and cognitive impairments similar to those seen in MS patients (Hobom et al. 2004; Wensky et al. 2005; Brown and Sawchenko 2007; Rasmussen et al. 2007; MacKenzie-Graham et al. 2009; Ziehn et al. 2010). Specifically, corpus callosum (CC) integrity in MS reflects demyelinating lesions, diffuse tissue damage, and abnormalities in neural connectivity, making it a potentially useful surrogate marker of clinically significant brain abnormalities (Boroojerdi et al. 1998; Warlop et al. 2008a,b; Ozturk et al. 2010). Thus, CC integrity was assessed by performing callosal conduction recordings and immunohistochemistry. Finally, as a predictive and longitudinal, *in vivo* functional biomarker to effectively assay the therapeutic efficacy of LQ, we measured rotorod motor performance (Tiwari-Woodruff et al. 2007). Rotorod performance is an established motor task used to evaluate balance and coordination aspects of motor function in rodents, and it has potential motor correlates with the Kurtzke Expanded Disability Status Scale (EDSS) used to measure disability in MS patients.

Our results demonstrate that both prophylactic and therapeutic treatment with LQ have significant beneficial effects in EAE mice. Specifically, LQ treatment attenuates EAE clinical disease and has immunomodulatory and neuroprotective effects that yield significant improvements in axon conduction and myelination. These effects correlate with significant improvement in rotorod motor performance. Our results support a potential neuroprotective, in addition to immunomodulatory effect of LQ treatment in inhibiting ongoing MS/EAE disease progression.

## Material and Methods

### Animals

Breeding pairs of Thy1-YFP mice (yellow fluorescent protein under the Thy1 promoter B6.Cg-Tg(Thy1-YFP)16Jrs/J) originally described by Feng et al. (2000) were purchased from Jackson Labs (stock number 003709) and were bred on the C57BL/6 background for more than five generation. In addition, breeding pairs of PLP_EGFP (proteolipid protein-enhanced green fluorescent protein) mice were a kind gift from Dr. Wendy Macklin (University of Colorado, Denver, CO). These mice were backcrossed to the C57Bl/6 strain for over 10 generation. All mice were bred in-house at the University of California, Los Angeles, animal facility. All procedures were conducted in accordance with the National Institutes of Health (NIH) and approved by the Animal Care and Use Committee of the Institutional Guide for the Care and Use of Laboratory Animals at UCLA.

### Laquinimod

Laquinimod (originally ABR-215062; RLB#054 M0004) was synthesized by Teva Pharmaceutical Industries, Ltd. The compound was dissolved in purified water and administered daily, by oral gavage, in a volume of 0.1 mL at 5 mg/kg or 25 mg/kg body weight. Treatment was initiated at post-immunization day 0 of EAE (pre-EAE+LQ) or after early (day 8; early post-EAE+LQ) to late (day 21; peak EAE+LQ) development of clinical manifestations and continued until the end of the experiment. Control mice received daily oral gavages of 0.10 mL purified water (EAE+vehicle).

### EAE

Active EAE was induced in 8-week-old male and female PLP_EGFP C57BL/6 mice (Tiwari-Woodruff et al. 2007; Crawford et al. 2010; Mangiardi et al. 2011). Specifically, active EAE was induced via immunization with 200 μg of myelin oligodendrocyte glycoprotein (MOG) peptide, amino acids 35–55, in combination with *Mycobacterium tuberculosis* in complete Freund's adjuvant (CFA) on post-immunization day 0 and 7. Additionally, mice were injected with pertussis toxin (PTX) (500 ng/mouse) on day 0 and 2. Mice were monitored and scored daily for clinical disease severity according to the standard EAE grading scale: 0, unaffected; 1, tail limpness; 2, failure to right upon an attempt to roll over; 3, partial hind limb paralysis; 4, complete hind limb paralysis; and 5, moribund. Within each treatment group, the mean clinical score was determined daily, thereby yielding the mean clinical score for that treatment group. Mice were followed clinically for up to 40 days after disease induction.

### Rotorod behavioral assay

Motor behavior was tested up to two times per week for each mouse using a rotorod apparatus (Med Associates, Inc., St. Albans, VT). Briefly, animals were placed on a rotating horizontal cylinder for a maximum of 200 sec. The amount of time the mouse remained walking on the cylinder without falling was recorded. Each mouse was tested on a speed of 3–30 rpm and given three trials for any given day. The three trials were averaged to report a single value for an individual mouse, and averages were then calculated for all animals within a given treatment group. The first two trial days prior to immunization (day 0) served as practice trials.

### Immune responses

Spleens were harvested during deep anesthesia with isoflurane prior to perfusion. Splenocytes were stimulated with the autoantigen MOG 35–55 peptide at 25 μg/mL. Supernatants were collected after 48 and 72 h, and levels of tumor necrosis factor (TNF)-α, interferon (IFN)-γ, interleukin (IL)-6, and interleukin (IL)-5 were determined by Searchlight (Aushon, Billerica, MA), as described previously (Tiwari-Woodruff et al. 2007).

### Histopathology and immunohistochemistry

Formalin-fixed coronal brain sections containing the CC, in addition to thoracic spinal cord were examined by immunohistochemistry using various series of cell type-specific antibodies, as previously described (Tiwari-Woodruff et al. 2007). In parallel, the CC was dissected and subjected to electron microscopy (EM) as previously described (Crawford et al. 2010). The following antibodies were used for immunohistochemistry to detect: axons: anti-neurofilament (NF200) (1:500, Millipore: MAB1621 and 1:1000, Sigma Aldrich, St. Louis, MO: N4142); astrocytes: anti-glial fibrillary acidic protein (GFAP) (1:1000, Millipore, Billerica, MA: 180063); oligodendrocyte (OL) progenitors (OLPs): anti-oligodendorcyte transcription factor 2 (olig2) (Millipore: AB9610) + anti-Ki67 (Millipore: AB9260); mature OLs: anti-CC1 (adenomatus polyposis coli, a mature OL marker) (1:1000, Gene Tex, Irvine, CA: GTX16794); PLP_EGFP fluorescence; myelin: anti-myelin basic protein (MBP) (1:1000, Millipore: MAB386, Abcam: 32760); T cells: anti-CD3 (1:1000, Abcam, Cambridge, MA: AB5690); microglia/macrophage/monocyte: leukocyte antigen marker anti-CD45 (1:500; Millipore: CBL1326, BD Biosciences, San Jose, CA: 550539), and damaged axons: anti-amyloid precursor protein (APP; Abcam: AB11132). The fluorescently tagged secondary antibody step was performed by labeling with antibodies conjugated to TRITC/Cy3 (Millipore: AP124C, AP132C), and Cy5 (Millipore: AP181S; AP187S). IgG-control experiments were performed for all primary antibodies and no staining was observed under these conditions. To assess the number of cells, a nuclear stain 4′,6-diamidino-2-phenylindole (DAPI; 2 ng/mL; Molecular Probes (Carlsbad, CA): D1306) was added for 15 min post-secondary antibody addition and prior to final washes. Sections were mounted on slides, allowed to dry, and coverslipped in fluoromont G (Fisher Scientific, Pittsburgh, PA). For EM, formalin- and glutaraldehyde-perfused brains were cut sagittally. The genu area of CC was identified under a dissecting scope and 4 mm^2^ blocks (from the mid-CC up to 1/3 splenium, corresponding to the CC area of plate 40–48 [Crawford et al. 2009b]) were carefully dissected. These blocks were further cut in 1 mm sections for Epon embedding.

### Microscopy and quantification

#### Stereological and *g*-ratio analysis

Immunostaining was quantified using unbiased stereology. Dorsal column was delineated using the drawing tool in Image J (Windows version 1.29 of NIH Image J; downloaded from http://rsbweb.nih.gov/ij/; Bethesda, MA). MBP, GFAP, CD3, and CD45 staining intensities were quantified within this region. NF200+ and MBP+ axons within a delineated region of the ventral column of thoracic spinal cord sections were quantified. All images (red, green, blue [RGB]) were converted to grayscale, split, and separated by color channel using ImageJ. To avoid experimenter bias, auto-adjustment of brightness and contrast, as well as threshold of staining signal, was carried out by NIH software. A Grid Plug-in (Image J) was used for counting points per area of interest. CC1+ OLs, olig2+ and Ki67+ OLPs, and CD3+ T cells lying within the CC or spinal cord dorsal column region were counted manually using 10× or 40× images and compared blindly between normal, EAE+vehicle, and EAE+LQ treated groups. Inflammatory cells were quantified by counting the number of CD45+ and CD3+ cells that were co-stained with DAPI+ nuclei in the delineated thoracic spinal cord dorsal column (and/or delineated CC). Myelin (MBP+) and astrocytes (GFAP+) were calculated as percent area intensity from the spinal cord dorsal column (Fig. 2) and delineated CC. Spinal cord axonal densities were calculated by counting the number of NF200+ cells in a 40× magnification image from the ventral funiculus white matter of thoracic cords where more coherent and similar diameter axons are present. Myelinated axonal densities were calculated by counting axons with a clear ring of MBP+ myelin staining around them. Damaged axons were calculated by counting APP+ axons.

For EM analysis, serial ultrathin sections of CC embedded in Epon were stained with uranyl acetate-lead citrate and analyzed as described in Crawford et al. (2010). Images at 3600× and 14,000× magnification were analyzed using Image J. A grid was used to count axons per area of interest. The ratio of axon diameter to total fiber diameter (*g*-ratio) was measured by dividing the circumference of an axon without myelin by the circumference of the same axon including myelin (Crawford et al. 2010). For most axons, two encounters were measured. At least 500 axons were analyzed per treatment group.

### Electrophysiological recording procedures

Brain slices (400 μm thick) corresponding approximately to plates 40–48 in the atlas of Franklin and Paxinos (2001) were used for electrophysiology recording, as previously described (Crawford et al. 2010). Compound action potential (CAP) recordings and axon refractoriness measurements were performed as previously described (Crawford et al. 2009a,b). Stimulation used for evoked CAP was constant current stimulus-isolated square wave pulses. Axon refractoriness is defined as the reduced excitability of an axon following an action potential. Axon damage can modify refractoriness and its measurement represents a diagnostic tool to measure axon health. To quantify refractoriness, the suppression of a second CAP response in paired stimulus trials is determined as previously described.

### Statistical analysis

Quantification of immunostaining results was similar to previous studies (Tiwari-Woodruff et al. 2007; Crawford et al. 2009b). To quantify immunostaining results, we used 2 sections (about 400 μm apart)/mouse from electrophysiology-recorded brains and 2 sections/mouse from perfused-fixed brains. There were *n* = 4 mice from electrophysiology + *n* = 4 mice that were perfuse fixed, with a total of *n* = 8 mice/per treatment group, for a total of 16 immunostained sections per treatment group. To quantify electrophysiology results from each treatment group: recordings from 2 caudal slices × 4 animals × 2 experiments = a total of 16 recordings were analyzed. For EM, 10 random caudal area fields/animal at 3600× and 14,000× magnification were used to quantify the “*g*-ratio.” Values are expressed mean ± SEM. Statistical analysis of mean values was carried out using one-way analysis of variance (ANOVA) and Friedman test (only for clinical scores) or Bonferroni's multiple comparison post-test. Differences were considered significant at the *P < 0.05 level. Statistical analyses were performed using MicroCal Origin (Northampton, MA) or Prism 4 (GraphPad Prism Software Inc., La Jolla, CA).

## Results

### LQ treatment decreases EAE disease severity equally in female and male mice

First, the dose range finding (DRF) of LQ effective in reducing the course of EAE clinical disease in male and female mice was confirmed. To visualize and characterize the effects of LQ on inflammation, demyelination, and axon degeneration, chronic EAE was induced in 8-week-old male and female PLP_EGFP (Mallon et al. 2002) or Thy1-YFP (Feng et al. 2000) transgenic mice bred on the C57BL/6 background. Thy1-YFP mice express spectral variants of green fluorescent protein at high levels in motor neurons, sensory neurons, and a subset of central neurons. Axons are brightly fluorescent and no expression is detectable in non-neural cells. PLP_EGFP mice show no fluorescence in neuronal cells or axons, but show high levels of green fluorescent protein in OL and myelin throughout the CNS.

**Figure 1:**
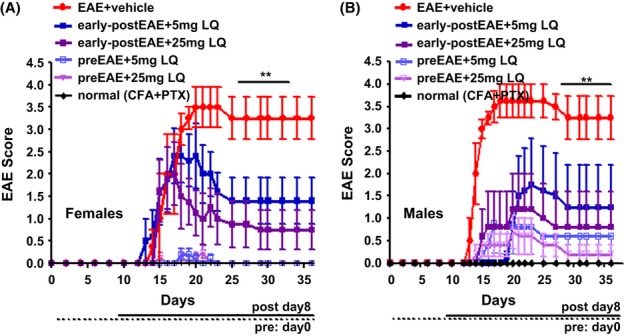
Laquinimod (LQ) treatment decreases EAE clinical disease severity equally in female and male mice. Eight-week-old female (A) and male (B) PLP_EGFP and Thy1-YFP C57BL/6 mice were immunized with MOG 35–55 peptide on post-inoculation day 0 and 7. Groups were treated daily, by oral gavage, with water (vehicle), 5 mg/kg LQ, or 25 mg/kg LQ beginning on day 0 (pre-EAE) or day 8 (early post-EAE). Mice were observed daily for clinical signs of EAE and scored on a five point scale. Pretreatment with 5 mg/kg and 25 mg/kg LQ entirely suppressed EAE clinical signs in both male and female mice. Early post-treatment with 5 mg/kg and 25 mg/kg also suppressed EAE clinical scores starting around day 20–25 (*n* = 10–12 mice/group; **P < 0.001, ANOVA Friedman test) as compared to EAE mice receiving vehicle alone. One of three representative experiments is shown.

Chronic EAE was induced using MOG 35–55 peptide (Mangiardi et al. 2011; Fig. 1A–B). Groups were treated daily, by oral gavage, with water (vehicle), 5 mg/kg LQ, or 25 mg/kg LQ beginning on post-immunization day 0 (pre-EAE) or day 8 (early post-EAE), prior to onset of clinical disease. A group of mice receiving CFA and PTX, but no MOG peptide, did not develop clinical disease (normal group). Vehicle-treated EAE mice developed a persistent, chronic disease course. A significant decrease in clinical disease development was shown in 5 mg/kg and 25 mg/kg pre-EAE LQ-treated female mice and they were similar to the normal group. Pre-EAE LQ-treated male mice showed a trend toward development of EAE symptoms but were not significantly different from the normal group. Even though early post-EAE treatment was started before development of symptoms, LQ treatment significantly decreased clinical disease in females and males by day 21. In both males and females, 25 mg/kg LQ was more effective in decreasing EAE clinical scores than 5 mg/kg LQ (Fig. 1).

### Early post-treatment with 25 mg/kg, but not 5 mg/kg, LQ reduces Th1 cytokine production by peripheral immune cells in EAE mice

**Figure 2:**
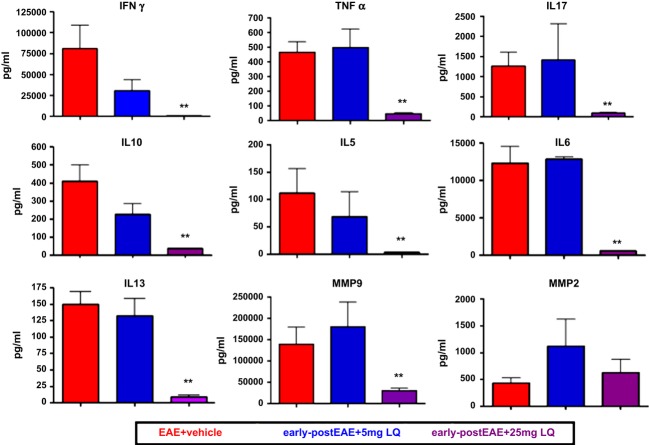
Post-treatment with 25 mg/kg, but not 5 mg/kg, laquinimod (LQ) reduces cytokine production by peripheral immune cells in EAE-induced female mice. EAE was induced as in Figure 1. Female mice from the 5 mg/kg and 25 mg/kg LQ early post-treatment groups were used for cytokine analysis. On day 36, cytokine production by MOG 35–55 peptide-stimulated splenocytes was determined. No significant change in cytokine levels was observed with 5 mg/kg LQ early post-treatment. There was an overall decrease in cytokine levels in the 25 mg/kg LQ early post-treated as compared to the vehicle-treated EAE group. Error bars indicate variability of cytokine values for splenocytes between individual mice within a given treatment group, with *n* = 5–8 mice/group. Data are representative of experiments repeated twice (**P < 0.001, *t-*test).

Previous studies have shown that LQ treatment redirected Type II monocyte differentiation, which was associated with reduced production of pro-inflammatory IL-6, IL-12/IL-23 (p40), and TNF, and increased production of anti-inflammatory IL-10 (Yang et al. 2004). Recently, marked decreases in IL-17, IL-5, and IL-10 levels and downregulation of pro-inflammatory cytokines IL-13, IFN-γ, and TNF-α were described in splenocytes from LQ-treated mice (Wegner et al. 2010). All of these studies assessed LQ treatment effects on cytokine levels during early EAE (post-immunization day 13), prior to the appearance of clinical symptoms. To address the consistent effects of LQ over time, cytokine levels of pre-EAE and early post-EAE LQ-treated mice were analyzed after day 36. Following final clinical score assessment on day 36 (Fig. 1), some mice were euthanized and their spleens were processed for splenocyte isolation and subsequent cytokine analysis. Splenocyte cytokine levels in the early post-EAE 5 mg/kg LQ group were similar to the vehicle-treated EAE group. However, decreased levels of IL-10, IL-5, and IL-6 and downregulation of Th1-pro-inflammatory cytokines (TNFα, IFN-γ, IL-13, and IL-6) in splenocytes from the early post-EAE 25 mg/kg LQ group, compared to vehicle-treated EAE mice, were observed. Matrix metalloproteinase (MMP-9) was significantly decreased in early post-EAE 25 mg/kg, but not 5 mg/kg, LQ-treated splenocytes as compared to vehicle-treated EAE splenocytes (Fig. 2). These results indicate that early post-treatment with 25 mg/kg LQ has anti-inflammatory effects on the peripheral immune system.

### Treatment with LQ attenuates inflammation and demyelination in spinal cords of chronic EAE mice

**Figure 3:**
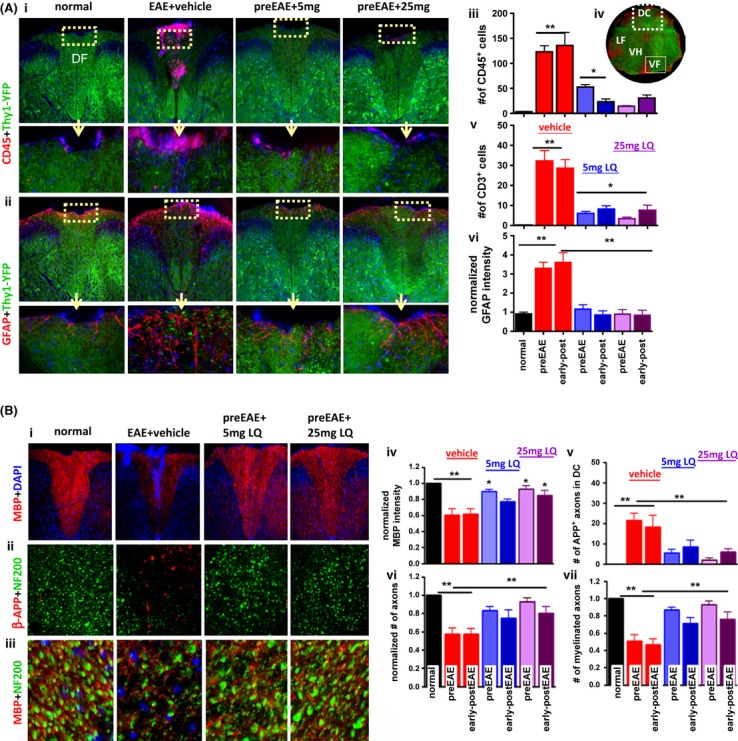
Laquinimod (LQ) treatment attenuates inflammation and demyelination in spinal cords of EAE mice. (A) Consecutive Thy1-YFP (green) thoracic spinal cord sections co-immunostained with CD45 (red, i) or GFAP (red, ii) at 10× magnification are shown from partial images (showing dorsal column; DC) from normal control, vehicle-treated EAE, 5 mg/kg LQ pretreated EAE, and 25 mg/kg LQ pretreated EAE mice at day 34. Spinal cords from vehicle-treated EAE mice show large areas of CD45+ and GFAP+ cell staining in the dorsal column as compared with normal controls, whereas 5 mg/kg and 25 mg/kg LQ pretreated EAE mice show only occasional CD45 and GFAP positivity. Consecutive sections from the same mice were also scanned at 40× magnification (within DC; designated by the area within the dotted line square) to show the morphology of CD45+ and GFAP+ cells. Number of CD45+/DAPI+ (iii), CD3+/DAPI+ (v), and GFAP+/DAPI+ (vi) cells per 400 μm^2^ within the DC were quantified from consecutive immunostained sections. Vehicle-treated EAE mice had a significant increase in CD45, CD3, and GFAP cells compared to healthy controls, whereas 5 mg/kg and 25 mg/kg LQ pre- and early post-treated EAE mice did not. A coronal cross section of thoracic spinal cord from a Thy1-YFP (green) and DAPI-stained (red) vehicle-treated EAE mouse imaged at 4× magnification (iv) is shown. The various regions of the spinal cord section are labeled as: DC, dorsal column; LF, lateral funiculus; VF, ventral funiculus; GM, gray matter. Statistically significant compared with normal controls (*P < 0.05; **P < 0.001; 1 × 4 ANOVAs; *n* = 6–8 mice/group). Data are representative of experiments repeated in their entirety on another set of EAE mice, with each of the treatment groups. (B) At post-immunization day 34, vehicle-treated EAE mice had reduced MBP (i), increased APP (ii), and decreased MBP+NF200 (iii) immunoreactivity, compared to normal controls, in the DC and VF of thoracic spinal cord sections imaged at 10× (i) and 40× (ii, iii) magnification. In contrast, 5 mg/kg and 25 mg/kg LQ pre- and early post-treated EAE mice showed relatively preserved MBP and NF200 staining. In addition, there was less APP+ immunoreactivity in LQ-treated groups as compared to vehicle-treated groups (ii). Upon quantification (iv–vi), MBP and NF200 immunoreactivity in the DC was significantly lower in vehicle-treated EAE mice as compared with normal mice, whereas LQ-treated EAE mice demonstrated no such significant decreases. Quantification revealed less MBP+ and NF200+ axons in the VF of vehicle-treated mice as compared to normal controls. LQ treatment reversed this trend; that is, there were less APP+ axons and more myelinated (MBP+ and NF200+) axons. Myelin density is presented as percent of normal. Statistically significant compared with normal controls (* *P* < 0.05; ** *P* < 0.001; 1 × 4 ANOVAs; *n* = 6–8 mice/group).

On post-immunization day 36 of EAE, another subset of mice from the experiment shown in Figure 5 was fixed by transcardial perfusion for histopathological evaluation and EM analysis. Spinal cord sections from Thy1-YFP and PLP_EGFP chronic EAE, vehicle-treated mice contained multiple areas with significantly decreased “green fluorescence,” indicative of neuronal and myelin pathology (Figs. 3 and 5). These areas of low green fluorescence were accompanied by inflammatory lesions with typical perivascular infiltration and accumulation of mononuclear cells, as previously seen (Mangiardi et al. 2011). CNS inflammation in vehicle-treated EAE mice includes activation of microglia/macrophages and increases in T cell numbers and cells of the monocyte lineage (Tiwari-Woodruff et al. 2007; Mangiardi et al. 2011). Consecutive thoracic (T1–T5) spinal cord sections were immunostained and imaged to show the dorsal column (Fig. 3A). Similar to previous observations, vehicle-treated EAE mice had numerous multifocal to coalescing inflammatory cell infiltrates that were positive for CD45, a pan-leukocyte marker which labels all infiltrating leukocytes, including T cells (Fig. 3A i–iii) and CD3+ T cells (Fig. 3A iv). Astrogliosis is also a prominent feature of the chronic and widespread adaptive CNS immune response in EAE and MS (Wu and Raine 1992; Liedtke et al. 1998). A significant increase in GFAP+ (a reliable astrocyte marker) immunoreactivity was observed throughout the gray and white matter of spinal cords from vehicle-treated EAE mice (Fig. 3A ii). Pre-EAE and early post-EAE LQ treatment significantly attenuated the reactive astrocyte response, as indicated by a significant decrease in GFAP staining intensity compared to vehicle-treated EAE mice (Fig. 3A ii–iv).

Consistent with demyelination, overall CD45+, CD3+, and GFAP+ cell infiltrates were associated with pallor and vacuolation in the white matter of spinal cord. Degree of myelin loss in the dorsal column of thoracic spinal cord was assessed using MBP immunostaining (Fig. 3B i). Extensive demyelination occurred at sites of cell infiltrates in vehicle-treated EAE mice as compared to normal controls. Significantly less demyelination occurred in nearly all LQ-treated spinal cords (Fig. 3B i). Quantification of demyelination in vehicle-treated EAE mice by analysis of MBP staining density in delineated dorsal columns revealed a ∼35% (*P* < 0.001) decrease in myelin density as compared with normal controls (Fig. 3B iv). In contrast, myelin staining was preserved in 5 mg/kg pre-EAE, 25 mg/kg pre-EAE, and 25 mg/kg early post-EAE LQ-treated dorsal columns, whereas the 5 mg/kg early post-EAE LQ-treated dorsal columns showed a trend toward increased MBP intensity but did not significantly differ from the vehicle-treated EAE group (Fig. 3B iv).

Considerable evidence now indicates that axonal injury is prominent in MS and EAE, and it suggests that axonal injury plays a prominent role in the progression of clinical signs (De Stefano et al. 2003). Here, potential axonal pathology was evaluated using NF200 and a prototypical marker of axonal damage, APP. In comparison with normal controls, EAE mice exhibited numerous APP+ axons and a significant reduction in the total number of NF200+ axonal profiles in the dorsal column (data not shown) and ventral funiculus (Fig. 3B ii). In comparison with vehicle-treated EAE mice, pre-EAE and early post-EAE LQ-treated mice exhibited an increase in number of NF200+ and significantly less APP+ axonal profiles (Fig. 3B ii, v, vi).

To assess the myelination status of NF200+ axons in the spinal cord of LQ-treated EAE mice, double immunostaining with antibodies to MBP and NF200 revealed relatively intact myelin rings (red) around axons (green) in normal and LQ-treated EAE mice (Fig. 3B iii, vii). Quantification of NF200 staining in the ventral funiculus revealed 49 ± 12% (*P* < 0.001) reduction in myelinated axons of vehicle-treated EAE mice compared to healthy controls. Significant increase in myelinated axons was observed in LQ-treated pre-EAE and early post-EAE groups as compared to vehicle-treated EAE group (Fig. 3B vi–vii).

### Treatment with LQ decreases EAE-induced callosal conduction and myelination deficit

**Figure 4:**
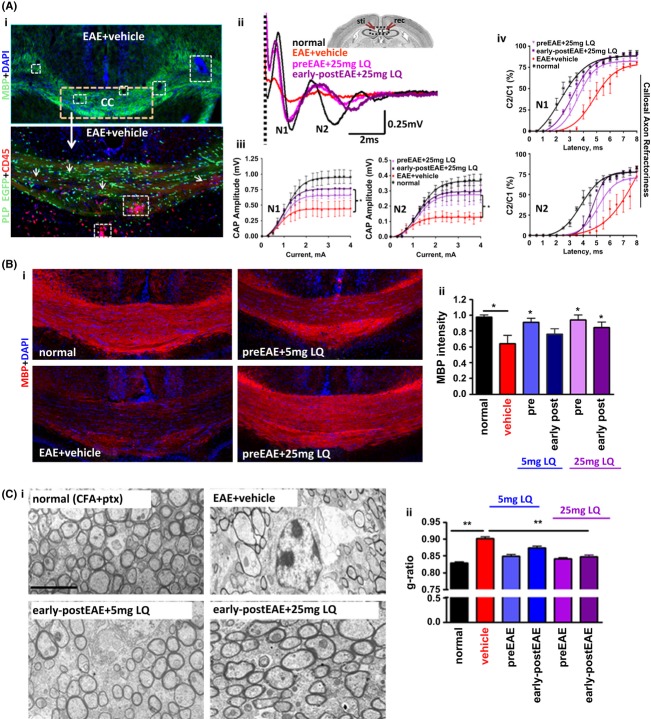
Treatment with laquinimod (LQ) decreases EAE-induced callosal conduction and myelination deficit. (A) Callosal lesions and demyelination during chronic EAE. (ii) Representative brain section containing CC white matter tracts from the PLP_EGFP group with late EAE was immunostained with MBP (green) + DAPI (red) and imaged at 4× magnification. Consecutive brain section from the same group immunostained with CD45+ (red) microglia/macrophage and imaged at 10× show many infiltrating lesions that are periventricular, around blood vessels (white dashed box) and scattered throughout the white matter, as well as decreased PLP_EGFP (green) cells and an increase in DAPI+ nuclei (also see Mangiardi et al. 2011). (ii, iii) Compound action potential (CAP) responses were recorded from slices with midline-crossing segments of the CC overlying the middorsal hippocampus. Stimulating (Sti) and recording (Rec) electrodes were each placed ∼1 mm away from midline. (CC, corpus callosum; Hip, hippocampus; S1, somatosensory cortex; M, motor cortex). Typical CC CAP from normal (black), vehicle-treated EAE (red), 25 mg/kg LQ pretreated EAE (magenta), and 25 mg/kg LQ early post-treated EAE (purple) brain slices evoked (at a stimulus of 4 mA) were recorded on post-immunization day 34. There was a decrease in N1 (fast conducting myelinated component) and N2 (slow conducting, mostly non-myelinated component) CAP amplitude in the vehicle-treated EAE group. Treatment with 25 mg/kg LQ during EAE inhibited the EAE-induced decrease in N1 and N2 CAP amplitude compared to vehicle treatment. Dashed vertical line represents CAP beyond the stimulus artifact (i). Quantification of N1 (ii) and N2 (iii) CAP amplitudes in the CC of vehicle-treated EAE and 25 mg/kg LQ pre- and early post-treatment groups was performed. Average C_2_/C_1_ ratios (obtained from plots of mean CAP amplitude) elicited by the second pulse in each paired stimulation (C_2_), divided by the CAP amplitude to single pulse stimulation (C_1_) (Crawford et al. 2009a,b, 2010) were fitted to Boltzmann sigmoid curves (iv–v). A rightward shift in curves for N1 and N2 shows decreased refractoriness in the vehicle-treated EAE group (*n* = 4). Callosal axons of LQ-treated EAE animals show a significant increase (a leftward shift in the curve compared to vehicle treatment) in refractoriness of N1 and N2. The interpulse interval (IPI) values (mean ± SD) of the N1 component are: normal = 2.2 ± 0.1 msec, vehicle-treated EAE 4.8 ± 0.2 msec, LQ pre-EAE = 3.7 ± 0.1 msec, and LQ early post-EAE = 3.1 ± 0.1 msec. The IPI values (mean ± SD) of the N2 component are normal = 3.2 ± 0.2 msec, vehicle-treated EAE = 8.8 ± 2.2 msec, LQ pre-EAE = 5.0 ± 0.2 msec, and LQ early post-EAE = 4.7 ± 0.2 msec. Statistically significant compared with normal controls at 2–4 mA stimulus strength (*P < 0.05; **P < 0.001; ANOVAs; Bonferroni's multiple comparison post-test; *n* = 6 mice/group). (B) Formalin-fixed brain sections collected from animals perfused on day 34 and brain sections from recorded slices were immunostained with anti-MBP (red) and imaged at 10× magnification. Vehicle-treated mice had reduced MBP immunoreactivity as compared to normal controls, while LQ-treated EAE mice showed relatively preserved MBP staining (i). Upon quantification, MBP immunoreactivity in CC was significantly lower in vehicle-treated EAE mice as compared to normal mice, while EAE mice pre- and early post-treated with LQ demonstrated no significant decreases (except for the 5 mg/kg LQ early post-treated EAE group). Myelin intensity is presented as percent of normal (*P < 0.05; ANOVAs; Bonferroni's multiple comparison post-test; *n* = 8–10 mice/group). (C) Representative electron micrographs of the CC from normal control, vehicle-treated EAE, and LQ early post-treated EAE mice show differential levels of axon myelination (i–ii). Compared to normal controls, the CC of vehicle-treated EAE mice show increased numbers of non-myelinated axons. Early post-treatment with 5 mg/kg and 25 mg/kg LQ resulted in a dramatic increase in myelinated axons as compared to vehicle-treated EAE mice. Photomicrographs are at 4800×. Scale bar is 1 μm. Axon diameter and fiber diameters of all axons in the field were measured to further quantify the degree of myelination. Axon diameter/fiber diameter (*g*-ratio) showed a significant increase in vehicle-treated EAE callosal axons and a dramatic decrease in LQ-treated EAE callosal axons (ii). Calculated *g*-ratio for callosal axons (mean ± SEM) is normal control = 0.83 ± 0.002, EAE+vehicle = 0.90 ± 0.004, pre-EAE+5 mg/kg LQ = 0.85 ± 0.004, pre-EAE+25 mg/kg LQ = 0.84 ± 0.005, early post-EAE+5 mg/kg LQ = 0.87 ± 0.04, and early post-EAE+25 mg/kg LQ = 0.85 ± 0.004. At least four mice from each group were analyzed and a minimum of 500 fibers were measured from each mouse (**P < 0.001; *P < 0.05; ANOVAs; Bonferroni's multiple comparison post-test).

We have recently shown that CNS structures (i.e., CC, hippocampus, and cerebellum) other than the spinal cord are negatively affected during EAE (MacKenzie-Graham et al. 2009; Ziehn et al. 2010; Mangiardi et al. 2011; Kumar et al. 2013), leading to sensory, motor, and cognitive impairments similar to those seen in MS patients. Callosal white matter tracts from EAE brains showed many periventricular infiltrating lesions around blood vessels (white dashed box) and scattered throughout the white matter accompanied by microglia/macrophage and reactive astrocyte accumulation and a marked decrease in PLP_EGFP^+^ OLs (Fig. 4A i; also see Mangiardi et al. 2011). The pathology observed in EAE callosal axons is similar to the pathology observed in white matter of EAE-induced spinal cords. Callosal axons play a significant role in interhemispheric transfer and integration of sensorimotor and cognitive information (Singer 1995). To characterize the functional consequences of CC neuropathology during EAE, CAPs were recorded in callosal axons (Fig. 4A). Coronal brain slices with midline-crossing segments of the CC, corresponding approximately to plates 45–48 in the atlas of Franklin and Paxinos (2001), were used for recordings (Fig. 4A i). Typical voltage traces showing two downward phases of the “N1” and “N2” CAP amplitude, likely representing fast depolarization from large, myelinated axons and slower depolarization from non-myelinated axons, respectively, are shown (Mangiardi et al. 2011). During EAE, both N1 and N2 CAP amplitudes were decreased to nearly 50% of normal (**P < 0.001, Fig. 4A i–iii). Treatment with 25 mg/kg LQ during pre-EAE and early post-EAE induced a significant increase in N1 and N2 CAP amplitude compared to vehicle treatment (*P < 0.05).

A recent study demonstrated that clinical, synaptic, and neuropathological defects in EAE mice were significantly attenuated by LQ treatment, indicative of potential neuroprotective effects (Ruffini et al. 2012). To investigate the effect of LQ on EAE-induced callosal axon deficits, changes in axon refractoriness were examined as previously described (Reeves et al. 2005; Crawford et al. 2009a). Axon refractoriness is defined as the reduced excitability of an axon following an action potential. Axon damage can modify refractoriness and its measurement represents a diagnostic tool to measure axon health. Rightward shifts in these curves correspond to increases in the refractory recovery cycle in axons and are indicative of functional deficit (Crawford et al. 2010; Mangiardi et al. 2011). In the normal group, the N1 component evoked by the second pair of pulses was 50% of the amplitude of a single pulse presentation at an interpulse interval (IPI) of 2.2 ± 0.1 msec (Fig. 4A iv). The IPI for the vehicle-treated EAE group had a slower response of 4.8 ± 0.2 msec, whereas 25 mg/kg LQ pre-EAE- and early post-EAE-treated callosal axons had a faster IPI of 3.7 ± 0.1 and 3.3 ± 0.2 msec, respectively. The IPI for the N2 component from vehicle-treated EAE mice (8.8 ± 2.2 msec) were significantly slower than those of normal mice (3.2 ± 0.2 msec). LQ treatment caused a significant recovery, trending to normal levels for pre-EAE (5.0 ± 0.2 msec) and early post-EAE LQ-treated animals (4.7 ± 0.2 msec; Fig. 4A v).

Electrophysiology-recorded post-fixed and non-recorded formalin-fixed brain slices were subjected to immunohistochemistry to assess myelin content. Similar to what was seen in spinal cord sections, and to previous results, a significant decrease in overall GFP fluorescence was observed in vehicle-treated PLP_EGFP mice (Mangiardi et al. 2011). Compared to normal controls, vehicle-treated EAE CC displayed a significant decrease in MBP+ and an increase in infiltrating DAPI+ nuclei centered on vessels (Fig. 4B). In contrast, 5 mg/kg pre-EAE and 25 mg/kg pre-EAE and early post-EAE groups showed a significant improvement in myelin (Fig. 4A, B). The 5 mg/kg earlypost-EAE LQ-treated group did not display a significant recovery in callosal myelination (*P < 0.05, Fig. 4B ii).

To assess the integrity of axon myelination, ultrastructure analysis of the CC was performed by EM. The ratio of the inner axonal diameter to the total outer diameter, termed the “*g*-ratio,” is widely utilized as a functional and structural index of optimal axonal myelination. High magnification images were used to calculate axon diameter and myelin thickness to generate mean *g*-ratios of all the myelinated and non-myelinated axons within a given field (Fig. 4C i, ii). At day 36, vehicle-treated EAE mice showed increased numbers of non-myelinated (49 ± 6%) and thinly myelinated callosal fibers compared to only 8 ± 4% non-myelinated axons in normal mice. The calculated *g*-ratio of vehicle-treated EAE CC was significantly higher than in normal controls. Number of non-myelinated axons decreased in EAE groups that were treated with LQ: 23 ± 5% for pre-EAE+5 mg/kg LQ; 18 ± 4% for pre-EAE+25 mg/kg LQ, 36 ± 7% for post-EAE+5 mg/kg LQ, and 23 ± 4% for post-EAE+25 mg/kg LQ as compared to the vehicle-treated group. In addition, the calculated *g*-ratio of LQ-treated groups was significantly lower than those of the vehicle-treated group (Fig. 4C i, ii). The mean calculated *g*-ratio of vehicle-treated EAE mice (0.90 ± 0.004) was significantly higher than for normal mice (0.83 ± 0.002). LQ treatment induced a decrease in axon demyelination and a potential increase in remyelination, leading to a significant recovery in *g*-ratio to near normal levels: early post-EAE+5 mg/kg LQ = 0.87 ± 0.004 and early post-EAE+25 mg/kg LQ = 0.85 ± 0.005 (**P < 0.001, ANOVA).

### Therapeutic treatment with 25 mg/kg LQ during peak EAE attenuates disease scores and suppresses pro-inflammatory cytokine production

**Figure 5:**
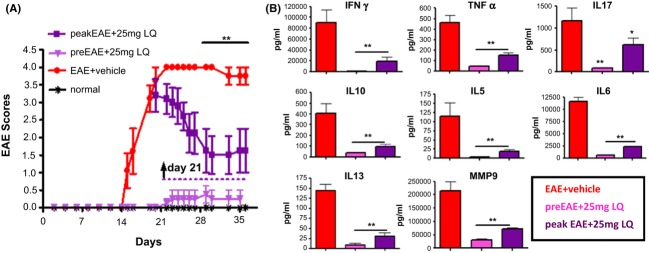
Therapeutic treatment with 25 mg/kg laquinimod (LQ) after onset of clinical EAE attenuates disease scores and suppresses cytokine production by peripheral immune cells. (A) PLP_EGFP and Thy1-YFP C57BL/6 female mice were administered 25 mg/kg LQ via oral gavages beginning on day 21 (purple squares; peak EAE) or day 0 (light purple triangle; pretreated) after initiation of active EAE or water (vehicle; red circle). Mice were scored using the standard EAE grading scale. EAE scores of mice pretreated with 25 mg/kg LQ were significantly improved throughout the duration of disease, whereas EAE mice treated with LQ beginning at peak EAE showed significant improvement after 7 days of continuous treatment, as compared to vehicle-treated EAE mice (**P < 0.001, ANOVA Friedman test). Normal mice did not show any disease and their clinical scores remained 0 throughout the experiment. Number of mice per treatment group: normal, *n* = 8; EAE+vehicle, *n* = 10; pre-EAE+LQ, *n* = 10; peak EAE+LQ, *n* = 15. Data are representative of experiments repeated three times. (B) EAE was induced and treatment was administered as in Figure 5A. On day 34, mice were euthanized and cytokine production by MOG 35–55 peptide-stimulated splenocytes was determined. Cytokine levels were each significantly reduced in 25 mg/kg LQ pretreated and with 25 mg/kg LQ post-treated groups. Error bars indicate variability of cytokine values for splenocytes between individual mice within a given treatment group, with *n* = 4 mice/group. Data are representative of experiments repeated twice (*P < 0.05, **P < 0.001, *t-*test compared to vehicle-treated group).

The 25 mg/kg dose of LQ was most efficient in ameliorating clinical disease during pre-EAE (post-immunization day 0) and early post-EAE (day 8). We wanted to test the effectiveness of LQ treatment in EAE mice exhibiting peak EAE clinical disease. Importantly, this period comprises significant demyelination and axon damage due to inflammatory episodes, and it is a translationally relevant scenario that more closely mimics therapy in MS patients, who receive treatment after a disease episode. Groups of PLP_EGFP and Thy1-YFP female mice were administered 25 mg/kg LQ beginning on day 21 during peak EAE (peak EAE), when the mean EAE clinical score was 3.0 (partial paralysis). EAE mice started on LQ treatment at day 0 (pre-EAE) and another group of EAE mice administered water (EAE+vehicle) were assigned as controls (Fig. 5A). LQ treatment started at peak EAE attenuated a further increase in clinical scores as compared to vehicle treatment. Eventually, there was a steady decline in the clinical disease score of this LQ-treated group, reaching ∼1.5 (**P < 0.001, *n* = 10 animals/group). Following final clinical score assessment on day 36 (Fig. 5A), some mice were euthanized and their spleens were processed for splenocyte isolation and subsequent cytokine analysis. Even when initiated near peak clinical disease, 25 mg/kg LQ treatment had a remarkable effect on cytokine levels. Similar to the 25 mg/kg pre-EAE LQ treatment group, splenocyte IL-10, IL-5, IL-13, and IL-6 cytokine levels were significantly decreased in the 25 mg/kg peak EAE treatment group. In addition, pro-inflammatory cytokines TNFα, IFN-γ, IL-6, and MMP-9 were downregulated in the 25 mg/kg peak EAE and pre-EAE treatment groups, as compared to the vehicle treatment group (*P < 0.05, **P < 0.001, Fig. 5B).

### Therapeutic 25 mg/kg LQ treatment initiated after onset of EAE clinical disease is neuroprotective

**Figure 6:**
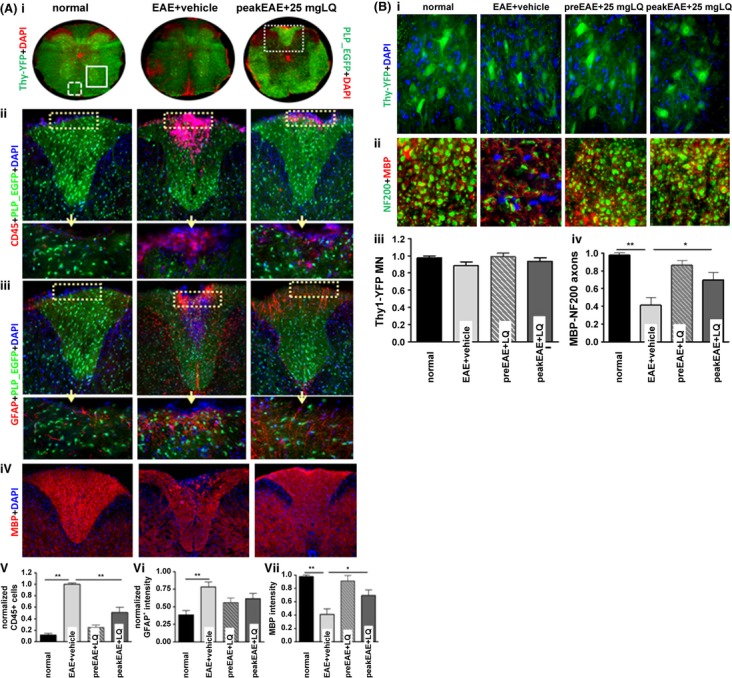
Therapeutic treatment with 25 mg/kg laquinimod (LQ) after disease onset reduces inflammation and axon demyelination in spinal cords of EAE mice. (A) Representative Thy1-YFP (normal and EAE+vehicle) and PLP_EGFP (peakEAE+25 mg/kg LQ) thoracic spinal cord sections (4× magnification) were co-stained with DAPI (red). White square boxes denote various areas of the spinal cord (i). Vehicle-treated EAE spinal cord had multifocal to coalescing infiltrates in the leptomeninges. No inflammatory nuclei are observed in normal controls, whereas peak EAE+25 mg/kg LQ shows less DAPI+ cell infiltrates in the lateral funiculus, dorsal column (DC), and ventral funiculus (i). Consecutive Thy1-YFP (green) thoracic spinal cord sections co-immunostained with CD45 (red, ii) or GFAP (red, iii) at 10× magnification are shown from partial images (showing DC) from normal control, vehicle-treated EAE, and peak EAE+25 mg/kg LQ mice at day 38 after disease induction. Vehicle-treated EAE spinal cords had large areas of CD45+ and GFAP+ cell staining in the DC as compared with normal controls, whereas peak EAE+25 mg/kg LQ mice showed a significant decrease in CD45 and GFAP positivity. Consecutive sections from the same mice were also scanned at 40× magnification (within DC, designated by the area within the dotted lines in the normal image) to show the morphology of CD45+ and GFAP+ cells (ii, iii). Number of CD45+/DAPI+ (v), GFAP+/DAPI+ (vi) cells per 400 μm^2^ and MBP immunoreactivity within the DC were quantified. Compared to normal controls, a significant increase was seen in vehicle-treated EAE mice but not 25 mg/kg LQ-treated EAE mice. The extent of demyelination was compared by immunostaining thoracic spinal cord sections with MBP. Myelin density is presented (*P < 0.05; **P < 0.001; 1 × 4 ANOVAs; *n* = 6–8 mice/group). Data are representative of experiments repeated in their entirety on another set of EAE mice. (B) The ventral horn and part of the anterior funiculus of immunostained thoracic spinal cord sections from normal controls, vehicle-treated EAE, and 25 mg/kg LQ-treated EAE Thy1-YFP mice euthanized on day 34 were imaged at 40× magnification. The number of Thy1-YFP+ motor neurons in the ventral horn did not change between groups, but the level of Thy1 immunoreactivity in vehicle-treated EAE neurons was significantly weaker. The 25 mg/kg LQ pre- or post-treatment in EAE mice recovered the level of Thy1-YFP fluorescence in these neurons (i). Consecutive sections immunostained with NF200 (green), MBP (red), and DAPI (blue) and imaged at 40× showed robust NF200 and MBP immunostaining in the anterior funiculus of normal controls and LQ pre- and post-treated EAE groups (ii). In contrast, sections from vehicle-treated EAE mice had decreased myelin and axonal staining. Quantification of Thy1-YFP motor neurons (MN) did not show significant differences in number between groups. Number of NF200+ axons that were also MBP+ decreased significantly in vehicle-treated EAE mice but not EAE mice pre- or post-treated with 25 mg/kg LQ. Statistically significant compared with normal controls (**P < 0.001; 1 × 4 ANOVAs; *n* = 6 mice/group).

On day 36 (from the experiment shown in Fig. 5A), a group of mice underwent transcardial perfusion in preparation for histopathological evaluation and EM analysis. Similar to previous observations, vehicle-treated EAE mice had numerous multifocal to coalescing inflammatory cell infiltrates that were CD45+ and GFAP+ combined with a significant decrease in MBP+ intensity in the spinal cord dorsal column (Fig. 6A i–iii). Treatment with 25 mg/kg LQ beginning on day 21 (peak EAE) significantly reduced the number of infiltrating CD45+ cells and GFAP+ intensity and induced a significant recovery of MBP+ staining intensity. Similarly, the 25 mg/kg pre-EAE LQ-treated group had minimal inflammation, decreased reactive astrocytes, and little demyelination (Fig. 6A i–iii). Quantification of CD45+ cells, GFAP+ cells, and MBP+ staining intensity showed a significant recovery in myelin density and a decrease in microglia/macrophages and astrocytes in peak EAE LQ-treated as compared to the vehicle-treated EAE group (Fig. 6 v–vii).

In addition to white matter pathology, several studies in both MS and EAE have indicated the presence of motor neuron pathology as an aspect of gray matter disease. Vogt et al. (2009) found a massive loss of lower motor neurons in MS patients compared to control subjects. This loss led to a decrease in compound muscle action potential amplitudes and motor unit numbers in MS patients compared to control subjects (Vogt et al. 2009). In previous studies, neuron atrophy, but not loss, in MOG-induced EAE of C57BL/6 mice has been indicated (Bannerman et al. 2005). By using Thy1-YFP transgenic mice, we were able to characterize and serially count motor neurons in the ventral horn of T1-T5 spinal cord sections. Spinal cords of vehicle-treated EAE mice showed similar numbers of motor neurons compared to normal controls. Analogous to previous studies, we consistently found significant decreases in neuronal processes and dendrites, as well as atrophied cell somas (Fig. 6B i). The pre-EAE LQ-treated group showed similar neuronal numbers, no sign of atrophy, and no significant process loss, in contrast with the vehicle-treated EAE group. Most remarkable was the effect of 25 mg/kg LQ treatment after peak EAE disease – no atrophy of motor neuron soma, no decrease in processes, and no dendrite decrease was observed compared to the vehicle-treated EAE group. Quantification of Thy1-YFP or NF200+ (not shown) neurons colabeled with DAPI showed no significant differences between groups (Fig. 6B i, iii).

Our previous work has shown a significant decrease in axon numbers and myelination in white matter of spinal cord by post-induction day 21 of EAE (Tiwari-Woodruff et al. 2007; Mangiardi et al. 2011). The effect of LQ treatment during peak disease on axonal pathology and demyelination was evaluated. Similar to previous observations, and in comparison with normal controls, vehicle-treated EAE ventral funiculus of thoracic spinal cords showed a significant decrease in myelinated (MBP+ and NF200+) axons (Fig. 6B ii). In comparison with the vehicle-treated group, 25 mg/kg LQ pre-EAE and peak EAE groups exhibited an increase in myelinated axon numbers (Fig. 3B ii, v, vi). Quantification of NF200 staining in the ventral funiculus revealed a 40 ± 12% (*P* < 0.001) reduction in vehicle-treated EAE mice as compared with healthy controls, whereas mice treated with LQ beginning at peak clinical disease showed a significant recovery to ∼70% myelinated axons of normal controls (Fig. 6B ii, iv).

### Therapeutic treatment with 25 mg/kg LQ after onset of EAE clinical disease attenuates EAE-induced callosal conduction deficits

**Figure 7:**
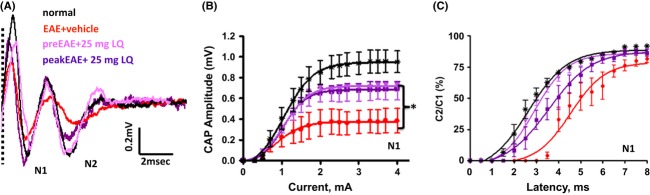
Therapeutic treatment with 25 mg/kg laquinimod (LQ) after onset of clinical EAE attenuates callosal conduction deficits and demyelination. (A) Compound action potential (CAP) responses were recorded from slices with midline-crossing segments of the CC overlying the middorsal hippocampus. Typical CC CAP from normal (black), vehicle-treated EAE (red), pre-EAE+25 mg/kg LQ (magenta), and peak EAE+25 mg/kg LQ (purple) brain slices on day 34 are shown. There is a decrease in N1 and N2 CAP amplitude in the vehicle-treated EAE group, as well as latency shift in the N2 peak. Both pre-EAE and peak EAE LQ treatment induced an increase in N1 and N2 CAP amplitude compared to vehicle alone, and normalization of N2 peak latency. (Dashed vertical line represents CAP beyond the stimulus artifact.) (B) Quantification of CAP amplitudes in the CC of vehicle-treated EAE mice showed a significant reduction in N1 amplitude. LQ pre- and post-treatment showed a significant improvement in CAP response. (C) Average C_2_/C_1_ ratio analysis of LQ-treated EAE callosal axons show a significant increase (a leftward shift in the curve) in refractoriness of N1 compared to vehicle treatment. The interpulse interval (IPI) values (mean ± SD) of N1 component are normal = 2.6 ± 0.1 msec, EAE+vehicle = 4.5 ± 0.2 msec, pre-EAE+LQ = 2.7 ± 0.1 msec, and peak EAE+LQ = 3.5 ± 0.1 msec. Similar trends were seen with the N2 IPI (data not shown). Statistically significant compared with normal controls at 2–4 mA stimulus strength (*P < 0.05; ** *P* < 0.001; ANOVAs; Bonferroni's multiple comparison post-test; *n* = 6 mice/group).

LQ treatment in EAE animals initiated after peak EAE disease attenuated axon damage and increased axon myelination. If this recovery is sufficient and functional, then it should afford improved axon conduction as compared to vehicle-treated EAE animals. CAP recordings of callosal axons were performed as described above (Crawford et al. 2010). A distinct improvement in peak N1 and N2 CAP amplitudes was observed in the LQ-treated pre-EAE group, as previously seen. Surprisingly, LQ treatment after peak disease resulted in a significant recovery in N1 and N2 CAP amplitudes, similar to pre-EAE LQ treatment and normal controls (Fig. 7). Quantification of N1 CAP amplitudes only is shown (Fig. 7B). To investigate the therapeutic treatment effect of LQ on EAE-induced axonal deficits, changes in axon refractoriness were estimated (Fig. 7C). The N1 IPI for the vehicle-treated EAE group had a slower response of 4.6 ± 0.1 msec, while LQ pre-EAE treated values of 2.7 ± 0.06 msec were similar to those of normal animals (2.5 ± 0.01 msec). There was significant recovery in the peak EAE LQ-treated group to 3.4 ± 0.1 msec (Fig. 7C). Similar trends were seen with N2 IPIs (data not shown).

### Therapeutic treatment with 25 mg/kg LQ significantly increases callosal myelination, decreases inflammatory cells, and increases survival of OL

**Figure 8:**
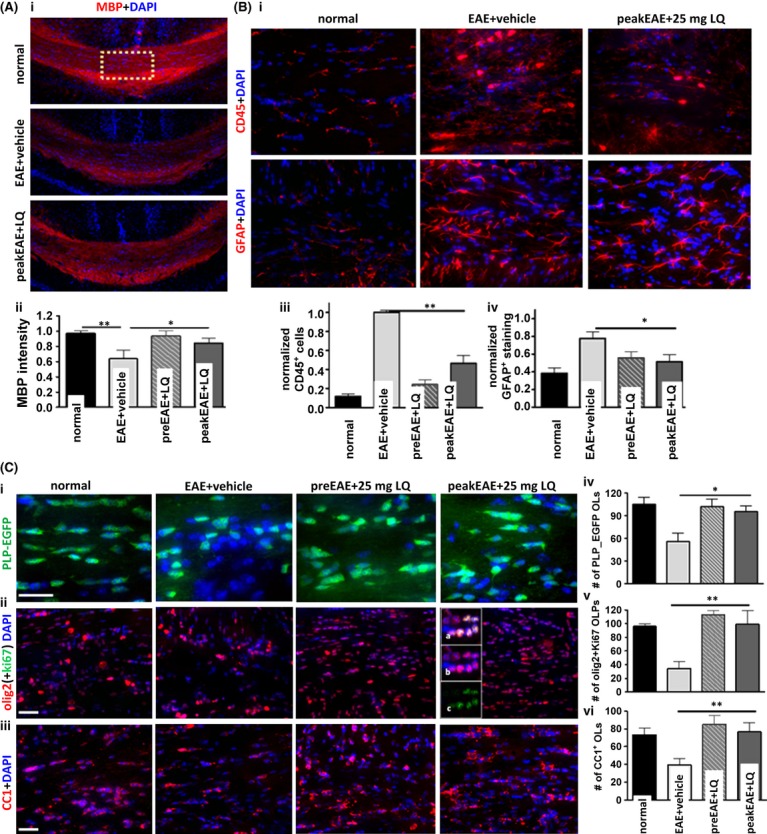
Prophylactic and therapeutic treatment with 25 mg/kg laquinimod (LQ) increases callosal myelination and oligodendrocyte survival but decreases inflammatory cells. (A) Formalin-fixed brain sections from mice euthanized at post-immunization day 34 were immunostained with anti-MBP (red) and imaged at 10× magnification. Vehicle-treated EAE mice had reduced MBP immunoreactivity as compared to normal controls, while LQ-treated EAE mice showed relatively preserved MBP staining (i). Myelin intensity is presented as percent of normal (*P < 0.05; ANOVAs; Bonferroni's multiple comparison post-test; *n* = 8–10 mice/group) (ii). (B) Consecutive brain sections were immunostained with CD45 (red, i) and GFAP (red, ii) and CC were imaged at 40× magnification. A marked increase in CD45 and GFAP immunoreactivity was observed in the vehicle-treated EAE group as compared to normal controls. Similar to a decrease in the pre-EAE LQ-treated group, peak EAE+25 mg/kg LQ brains had a significant decrease in CD45 and GFAP immunoreactivity. This was confirmed by counting the number of CD45+/DAPI+ (iii) and GFAP+/DAPI+ (iv) cells per 400 μm^2^ within the dorsal column. (C) To investigate the potential role of oligodendrocytes (OLs) in LQ treatment-induced preservation of myelin, brain sections from PLP_EGFP mice were colabeled with DAPI (blue) and olig 2, a marker of proliferating OL (co-immunostained with the proliferation marker Ki67, green; only shown in inset panel C); (i), as well as OL marker APC/CC1 (red, ii). Representative CC sections from normal controls, vehicle-treated EAE, 25 mg/kg LQ pre- and post-treated EAE mice sacrificed on day 34 and imaged at 40×. Compared to the CC of vehicle-treated EAE mice, the number of PLP_EGFP cells (i), proliferating OL (ii), and OL (iii) were significantly increased in LQ-treated EAE groups. Quantification of the number of PLP_EGFP (iv) costained olig2 + Ki67 cells (v) and CC1+ cells (vi) per 400 μm^2^ indicated a significant decrease in vehicle-treated EAE mice compared to normal controls. LQ treatment caused a significant increase in all three as compared to vehicle-treated EAE mice (*P < 0.05; **P < 0.001; ANOVAs; Bonferroni's multiple comparison post-test; *n* = 8–10 mice/group).

Electrophysiology recorded, post-fixed brain, and formalin-fixed brain sections were used to assess LQ-induced changes in immune cells, OLs, and axon myelination in animals treated after peak EAE clinical disease. A significant decrease in myelin staining intensity was observed in the vehicle-treated EAE group, similar to what was seen before. A significant increase in myelin fluorescence intensity that was not significantly different from the pre-EAE LQ-treated EAE group was observed in the peak EAE LQ-treated group (Fig. 8A i, ii). Similar to previous observations, CNS CD45+ immune cells and GFAP+ reactive astrocytes were significantly increased in vehicle-treated EAE animals (Fig. 8B iv). Peak EAE LQ treatment induced a significant decrease in the number of CD45+ and GFAP+ cells (similar to pre-EAE treatment) as compared to the vehicle-treated EAE group (Fig. 8B iv).

Increased myelin intensity in LQ-treated EAE mice can be attributed to an increase in survival of oligodendrocyte progenitors (OLP) or decrease in OL apoptosis. A direct PLP_EGFP+ cell count comparison of various treatment groups revealed a significant decrease in numbers of PLP_EGFP cells in vehicle-treated EAE mice. In contrast, pre-EAE and peak EAE LQ-treated mice showed increased numbers of PLP_EGFP cells. Morphological analysis of PLP_EGFP cells showed an improvement cell soma and cell processes in LQ-treated versus vehicle-treated EAE CC (Fig. 8C i). Because PLP_EGFP positivity does not distinguish between proliferating and differentiating OL, consecutive brain sections were co-immunostained with the proliferation marker Ki67 and olig2, as well as a marker of mature OL, CC1. In pre-EAE and peak EAE LQ-treated animals, a significant increase in proliferating OL (colabeled olig2+ and Ki67+ cells) was observed. In addition, CC1+ cell numbers were recovered in the peak EAE LQ-treated group as compared to the vehicle-treated EAE group (Fig. 8C i–iv).

### Therapeutic treatment with 25 mg/kg LQ significantly decreases EAE-induced motor deficit as measured by rotorod motor performance

**Figure 9:**
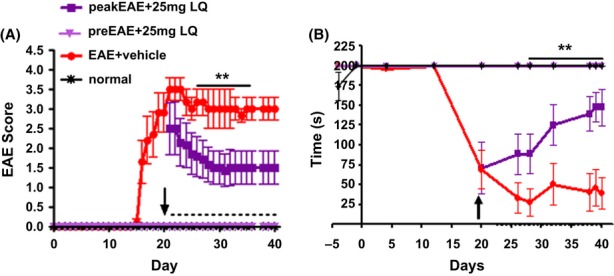
Prophylactic and therapeutic treatment with 25 mg/kg laquinimod (LQ) significantly decreases EAE-induced motor deficit, as measured by rotorod motor performance. (A) In a separate experiment, PLP_EGFP C57BL/6 female mice were given 25 mg/kg LQ via oral gavage beginning on day 20 (purple squares; peak EAE) or day 0 (light purple triangle; pre-EAE) after initiation of active EAE. Another group of EAE mice was administered water (vehicle) by oral gavage (red circle). Mice were scored using the standard EAE grading scale. EAE scores of LQ pretreated mice were significantly improved throughout the duration of disease, whereas LQ post-treated mice showed significant improvement after 7 days of continuous treatment compared to vehicle-treated EAE mice. Normal mice did not show any disease and their clinical scores remained zero throughout the experiment. (**P < 0.001; ANOVA; Friedman test; *n* = 10 mice/group). (B) To assess the clinical significance of LQ treatment during pre-EAE and peak EAE, mice from Figure 9A were subjected to a motor test frequently used to assess spinal cord injury-rotorod performance. Vehicle-treated EAE mice (red) demonstrated an abrupt and consistent decrease in time (seconds) they were able to remain on the rotorod beginning at day 15 after disease induction, and this disability remained throughout the observation period. When a group of vehicle-treated EAE mice were switched to 25 mg/kg LQ treatment at day 20 (purple square), the time point at which the mean EAE score reached ∼2.5, disability continued but was less severe. However, later, at day 30–40, the LQ-treated peak EAE group exhibited significant recovery. Normal (black) and 25 mg/kg LQ pretreated group (magenta) did not show any motor deficit. (**P < 0.05; ANOVA; *n* = 10).

To assess the functional significance of LQ treatment during pre-EAE and peak EAE, EAE mice in a separate experiment were subjected to a motor test frequently used to assess spinal cord injury – rotorod motor performance. EAE was induced in PLP_EGFP mice and animals were ultimately organized into the following treatment groups: vehicle, pre-EAE LQ, or peak EAE LQ. 25 mg/kg LQ was administered to one group of mice beginning on day 0 (pre-EAE). When clinical disease in vehicle-treated EAE mice reached ∼2.5–3 at day 20, treatment with 25 mg/kg LQ was initiated (peak EAE). EAE scores of pre-EAE 25 mg/kg LQ-treated mice were significantly improved throughout the duration of disease. Contrastingly, the 25 mg/kg peak EAE LQ group showed significant improvement only after 7 days of continuous treatment, as compared to vehicle-treated EAE mice. Normal mice did not show any signs of disease and their clinical scores remained 0 throughout the experiment (Fig. 9A).

Vehicle-treated EAE mice demonstrated an abrupt and consistent decrease in the time (seconds) they were able to remain on the rotorod beginning at day 15 after disease induction, and this disability remained throughout the observation period. When the average EAE score reached ∼2.5, vehicle-treated EAE animals that were switched to 25 mg/kg LQ treatment (at day 20) initially showed significant motor disability. However, within 5–7 days after initiation of treatment, motor disability was less severe. By day 30–40, the LQ-treated EAE group exhibited significant recovery in motor function (**P < 0.05, ANOVA, *n* = 10 animals/group; Fig. 9B).

## Discussion

Our study demonstrates that LQ treatment is effective in ameliorating EAE clinical disease even after EAE-induced inflammation, axon damage, and demyelination have been initiated. We analyzed callosal white matter integrity in addition to spinal cord, as the CC in MS reflects demyelinating lesions, diffuse tissue damage, and abnormalities in neural connectivity, making it a potentially useful surrogate marker of clinically significant brain abnormalities (Boroojerdi et al. 1998; Warlop et al. 2008a,b; Ozturk et al. 2010). Specifically, LQ inhibits ongoing demyelination and axon damage in the spinal cord and CC of EAE mice, leading to substantial recovery of callosal axon conduction, a functional indicator of axon myelination and neuroprotection. In addition, LQ treatment reverses ongoing motor deficit as measured using standard EAE clinical scoring and rotorod motor performance. While many reports focus on the prevention or inhibition of early EAE symptoms using LQ, ours is the first study to show distinct improvement in axon myelination, axon conduction, and motor function as a result of LQ treatment in pre (day 0), early post (day 8), and peak (∼day 21) EAE mice.

We took advantage of PLP_EGFP and Thy1-YFP transgenic mice to study the direct effects of LQ treatment on neurodegeneration and demyelination in EAE. LQ treatment significantly attenuated the loss of GFP fluorescence in neurons, axons, and OLs of the CNS. Most remarkable was the increased axon remyelination, axon conduction, and the significant recovery in proliferating and mature OL numbers of EAE animals treated with LQ after peak disease. This recovery correlated with LQ-mediated suppression of cytokine production and reduction in infiltrating immune cells in the CNS. Previous studies have also shown LQ-induced neuroprotection in EAE CNS (Ruffini et al. 2012), which correlates with suppression of cytokine production and reduction in infiltrating immune cells (Yang et al. 2004; Wegner et al. 2010; Aharoni et al. 2012; Bruck et al. 2012). In the present study, peripheral cytokine levels determined after post-immunization day 30 of EAE following 25 mg/kg LQ treatment were similar to levels observed at day 13 (i.e., downregulation of pro-inflammatory cytokines IL-13, IL-17, IFN-γ, and TNF-α (Wegner et al. 2010). In addition, there was a significant decrease in IL-4, IL-5, and IL-6 cytokines. Our findings of LQ-induced reduction in IL-10 levels in EAE mice are similar to Wegner et al. (2010) and contrast the increase in IL-10 levels reported by Yang et al. (2004). MMP have a crucial function in the migration of peripheral inflammatory cells into the CNS and levels of MMP-9 are elevated in MS and EAE. The 25 mg/kg LQ treatment during pre-EAE and peak EAE significantly decreased MMP-9 levels. Taken together, our findings suggest that LQ protects myelin and axons by decreasing pro-inflammatory cytokines and impairing the migratory capacity of inflammatory cells.

Reactive astrogliosis is a prominent feature of the chronic and widespread adaptive immune inflammation of the CNS that occurs during EAE and MS (Eng 1985). Reactive astrocytes are responsible for the production of pro-inflammatory molecules (e.g., cytokines, chemokines, growth factors, nitric oxide), growth-inhibitory molecules, and increased production of NFκB-dependent pro-inflammatory molecules. These molecules are detrimental to oligodendrocyte survival, remyelination, and functional recovery (Brambilla et al. 2009; Chang et al. 2012). A recent study using cuprizone diet-induced demyelination in mice demonstrated that LQ treatment reduces the inflammatory response in astrocytes by interfering with astrocytic NF-κB activation *in vivo* and *in vitro* (Bruck et al. 2012). Similarly, in the present study, LQ treatment significantly decreased the number of reactive astrocytes in the spinal cord and CC of pre-, early post-, and peak-EAE mice. Such findings point to an immunomodulatory mechanism through which LQ acts to uphold OL health and blunt demyelinating effects of EAE.

Notwithstanding, LQ's mechanisms of action are still being investigated. LQ is a small molecule that passively enters all cell types. A direct effect of LQ on astrocytes via modulation of NF-κB to reduce inflammatory response, or an indirect effect on various cell types and increase in growth factors, may contribute to cell survival, axon myelination, and neuroprotection in EAE and MS (Aharoni et al. 2012; Bruck et al. 2012; Thone et al. 2012). One of the many neurotrophic factors essential for neuronal function and myelination is BDNF. During EAE, BDNF mRNA and protein are significantly decreased, specifically in neurons, though higher immunoreactivity is observed in reactive astrocytes and immune cells around the lesions (Linker et al. 2010). A recent study showed that LQ treatment during EAE restores BDNF expression to normal levels (Aharoni et al. 2012). Similarly, a significant elevation of serum BDNF levels in relapse remitting MS (RRMS) patients receiving LQ therapy was observed (Thone et al. 2012). BDNF/tyrosine kinase receptor B (TrkB)-stimulated intracellular signaling is critical for neuronal survival, morphogenesis, plasticity, normal OL development, and axon myelination (Hetman et al. 1999; Linker et al. 2009; Numakawa et al. 2010; Vondran et al. 2010; Xiao et al. 2010; VonDran et al. 2011). LQ treatment-induced increase in BDNF and modulation of immune and astrocyte cell signaling could be responsible for improvement in OL proliferation/and survival that leads to improved axon remyelination and neuroprotection observed.

In summary, LQ treatment asserts a significant positive effect on axon myelination during EAE. LQ treatment caused a significant improvement in clinical scores even after peak EAE disease, when significant axon demyelination, axon damage, and OL apoptosis have already been established (Tiwari-Woodruff et al. 2007; Mangiardi et al. 2011). Rotorod motor performance by EAE mice can be correlated to the motor aspect of EDSS scores in MS patients, and it can be an essential biomarker for therapeutic MS drugs. At peak disease, EAE animals are unable to remain on the rotorod due to significant motor deficits. Upon treatment with LQ (before onset of or after peak disease), EAE animals exhibited significant motor improvement. Motor performance improvement correlated with improved callosal conduction and cell survival in the CNS. These findings strongly suggest that LQ treatment has significant and functional beneficial effects on neuron survival, decreasing axon damage, improving survival and proliferation of OLs, and increasing myelination/remyelination. A recent study emphasized that endogenous remyelination of the cerebral cortex can occur in individuals with MS, regardless of disease duration (Chang et al. 2012). With this in mind, LQ treatment could be beneficial to both RR and progressive forms of MS.
